# Positioning of the Central Venous Catheter for Hemodialysis Using Wireless Intracavitary ECG: A Case Series and Narrative Review of the Literature

**DOI:** 10.3390/medsci13020039

**Published:** 2025-04-02

**Authors:** Simone Gianazza, Cristina Valli, Stefano Mangano, Arline Vechiu, Monica Breda, Laura Composto, Clara Claudia Sardo, Camilla Ariti, Andrea Rizzi

**Affiliations:** 1 Department of General Surgery, Ospedale L Galmarini Tradate—ASST Settelaghi, 20149 Tradate, Italy; andrea.rizzi@asst-settelaghi.it; 2 Department of Accident and Emergency, Ospedale L Galmarini Tradate—ASST Settelaghi, 20149 Tradate, Italy; cristina.valli@asst-settelaghi.it (C.V.); monica.breda@asst-settelaghi.it (M.B.); laura.composto@asst-settelaghi.it (L.C.); claraclaudia.sardo@asst-settelaghi.it (C.C.S.); camilla.ariti@asst-settelaghi.it (C.A.); 3 Department of Nephrology and Dialysis, Ospedale L Galmarini Tradate—ASST Settelaghi, 20149 Tradate, Italy; stefano.mangano@asst-settelaghi.it; 4 Department of Medicine and Innovation Technology, University of Insubria—ASST Settelaghi, 21100 Varese, Italy; avechiu@studenti.uninsubria.it

**Keywords:** central venous catheter, central venous access, vascular access, hemodialysis, intracavitary ECG, tip location

## Abstract

This study aimed to evaluate the practicality and feasibility of using intracavitary electrocardiography to confirm the proper placement of a central venous catheter for hemodialysis. Central venous catheters are typically placed using an echo-guided technique based on anatomical landmarks, followed by X-ray confirmation. Anesthesiology guidelines recommend evaluating the intracavitary electrocardiogram during the procedure to verify the correct CVC placement. This study involved 11 patients without rhythm disturbances, in whom a central venous catheter was placed in the right internal jugular vein at our institute in 2024. The patient’s electrocardiogram was analyzed using the MAGELLANO^®^ (Italy) device to identify changes in the P wave or QRS complex, which confirmed the CVC’s correct placement at the right cavoatrial junction. Thoracic ultrasound was used to identify the right internal jugular vein and exclude iatrogenic pneumothorax. A subsequent chest X-ray was performed to further confirm the correct placement. In addition, a non-systematic review of the most recent literature on this topic was conducted using the Database PubMed—United States National Library of Medicine. Chest X-ray consistently verified the correct placements identified by ECG-IC, with no post-procedure complications. ECG-IC is a straightforward, viable, and cost-effective technique with high sensitivity when administered by properly trained professionals. This approach, combining ultrasound-guided CVC placement in the right internal jugular vein and intracavitary ECG monitoring, can omit X-ray control in more than 90% of cases.

## 1. Introduction

An increasing number of patients annually require haemodialysis due to renal failure, end-stage kidney disease, and other clinical conditions that necessitate the placement of a durable vascular access, such as an arteriovenous fistula or graft, to facilitate dialysis treatments [1]. The rates of both chronic kidney disease and end-stage kidney disease show a global increase, leading to a growing population of patients who require dialysis. This means there is an ever-rising number of patients being assessed for, and commencing on, haemodialysis to manage their renal failure and maintain their health and quality of life.

Historically, the arteriovenous fistula (AVF) has been regarded as the gold standard vascular access for hemodialysis; however, in a growing number of cases, the process now begins with temporary or tunneled cuffed catheters. Traditionally, there has been an associated survival benefit for patients on hemodialysis who have an arteriovenous fistula compared to those using a central venous catheter. This survival advantage is attributed to the superior hemodynamics and reduced risk of infection associated with the arteriovenous fistula approach to vascular access [2].

Central vein catheterization for hemodialysis was first reported by Erben in 1969 [3]. Central venous catheters are indwelling devices inserted into large veins, facilitating venous access and providing benefits for a diverse range of patients in various clinical settings, including but not limited to emergency departments, intensive care units, radiologic settings, and long-term medication management.

Central venous catheters have become essential for the management of critically ill patients, allowing for the administration of medications, fluids, hemodynamic monitoring and in several cases, for hemodialysis [4].

In the medical literature, a variety of indications have been identified that may justify the use of a central venous catheter, such as the need for administration of fluids, medications, or parenteral nutrition, chemotherapy for oncologic patients, as well as for hemodynamic monitoring and blood sampling; however, when used for dialysis purposes the following indicators can be present [5,6]:

▪ While awaiting the creation or maturation of an arteriovenous fistula;
▪ While anticipating a kidney transplant;
▪ When an AVF is contraindicated due to medical reasons, such as terminal heart failure;
▪ When a suitable site for an AVF is unavailable.

Despite the risk association of central venous catheters with infections, sepsis episodes, and higher mortality rates in patients requiring hemodialysis, a “fistula-first” approach was previously advocated. However, these patients are often older, frailer, and have more comorbidities, so the evidence supporting the survival benefit of an arteriovenous fistula over a CVC in this older, comorbid hemodialysis population has become less clear. Furthermore, older patients with comorbidities such as vascular disease frequently present unique challenges that can complicate the creation and maturation of an AVF. For instance, maturation rates may be lower, and the competing risk of death may lead to mature AVFs that are never used for dialysis, necessitating the use of CVCs as a practical and preferable alternative [2]. Consequently, the use of catheters among hemodialysis patients has increased by 1.5 to 3 times across many countries [7]. The paradigm has shifted from “fistula first” to “right access for the right patient at the right time” [2]. Clinicians must consider possible complications but must rely on the risk–benefit ratio between AVF and CVC in critically ill patients undergoing hemodialysis [7].

In recent times, thanks to advancements in manufacturer materials, improved hemocompatibility, enhanced performance with increased flow rates, and reduced recirculation [8], these catheters are frequently used as permanent access devices [9]. The persistent and widespread use of hemodialysis central venous catheters underscores their indispensable role in the delivery of modern medical care [7]. Various vascular access approaches have been explored over the years. A higher approach technique required the cannulation of the subclavian vein. However, this site was eventually abandoned due to a higher incidence of thrombosis and stenosis. Angiographic studies of the subclavian vein demonstrated complete lumen occlusion just a few days after catheter insertion, with hemodynamic consequences involving the venous drainage of the limb [1].

According to the latest research, the right internal jugular vein (RIJV) is the recommended site for the procedure [5,10,11].

Theoretically, each of these techniques carries the risk of arterial and venous complications, which can arise not only from the insertion and removal maneuvers that may result in embolism and migration, but also from mechanical and pharmacological treatments leading to catheter obstruction and the spread of microemboli. The complications of those approaches may include pneumothorax, hemothorax, hemomediastinum, and atrial perforation. Additionally, the risk of infection must be taken into account [8,12].

Compared to subclavian cannulation, the right internal jugular vein access reduces the risk of pneumothorax but carries a higher risk of accidental puncture of the carotid artery, the thyrocervical trunk, and the subclavian artery [8]. Additionally, jugular vein cannulation does not impact the venous drainage of the arm [13,14].

Various techniques have been described to achieve venous cannulation [5], including surgical cutdown, percutaneous Seldinger technique guided by anatomical landmarks, and percutaneous Seldinger technique under ultrasound guidance.

The RIJV is located within the sternocleidomastoid triangle of Sedillot, bounded by the medial and lateral heads of the sternocleidomastoid muscle with the clavicle as the base. The vein runs under the apex of the triangle, laterally to the carotid artery. However, even with accurate knowledge of neck anatomy, the landmark technique may be challenging for clinicians [13], as it is often difficult to access the RIJV using only anatomical landmarks, especially in patients who are obese, have neck swelling, or have had prior surgery or catheter placement [3].

This technique that uses blind insertion has a failure rate between 7 and 19% and may require multiple attempts until successful [13]. Given these findings, ultrasound (US) guidance represents the optimal choice, providing real-time imaging of the anatomy. The US ensures the vein is localized prior to cannulation, delineating the anatomy [10]. The vein can be easily distinguished from the carotid artery due to the vein’s larger diameter, and lateral and anterior positioning. The lack of pulsation, compressibility under pressure, and increased lumen induced by the Valsalva maneuver are additional distinguishing features [11,12,13].

Jugular vein catheterization under US guidance achieves a 100% success rate and can be applied in various clinical settings, including at the bedside, in radiology suites, in surgical theaters, and in both high-acuity and intensive care environments [9,10,11].

After central venous access, verifying the correct tip location is a critical step. Central venous catheter misplacement is a prevalent complication, defined as the catheter tip not being in the optimal position. The severity of CVC misplacement varies, with the most common form being simply malpositioning of the tip itself, and the rarer but more serious complication of extravascular placement. The mechanisms underlying CVC misplacement appear to be multifactorial. Existing research indicates that certain patient characteristics, such as obesity or large breasts, can promote catheter tip migration and increase the risk of improper positioning. Despite these factors, it is important to note that misplaced CVCs have been documented in a wide range of anatomical locations and across diverse patient body types.

So a precise position check is necessary to avoid complications related to the tip’s position. Tips positioned too low are associated with arrhythmias, while tips positioned too high may result in malfunction, fibroblastic sleeve formation, or thrombosis. The ideal zone for the tip of a central venous access (CVA) device is just above or just below the cavoatrial junction, an area situated between the lower third of the superior vena cava (SVC) and the upper portion of the right atrium (RA) [15].

The tip position can be assessed during the procedure using various techniques, including fluoroscopy, transthoracic echocardiography, transesophageal echocardiography, and intracavitary electrocardiography. In terms of accuracy and precision, no method can compete with Trans-Esophageal Echocardiography, at least as far as catheters inserted through the superior vena cava; however, it is expensive, invasive, and difficult to apply. Trans-Thoracic echocardiography is accurate only when the tip is visualized in the right atrium. Fluoroscopy is openly discouraged by current guidelines because it is inaccurate, potentially harmful to the patient and operator due to exposure to ionizing radiation, expensive, and logistically problematic [16].

In the past, chest radiography was routinely performed after subclavian or RIJV cannulation, initially to verify the correct catheter tip positioning, and subsequently to exclude potential complications such as pneumothorax and hemothorax [17]. X-ray imaging was recommended during and at the conclusion of the procedure [3]. The tracheal carina serves as an optimal radiographic landmark, with the cavoatrial junction (CAJ) typically situated approximately 3–5 cm below, while the lower third of the SVC resides within 3 cm inferior to the carina [18].

Currently, a more effective approach for detecting the catheter tip location involves the use of right atrial echocardiography, as introduced by Serafini in 1989 [3]. Based on the electrophysiological principles of electrocardiography, the morphology of the P wave reflects the electrical activity of the atrium and the position of the intracavitary electrode relative to the sinoatrial node. This technique involves connecting the catheter to an ECG recorder and monitoring the DII lead. As the catheter approaches the sinoatrial node, the RA P wave exhibits a negative deflection and a significant increase in amplitude [19,20]. Once the catheter tip surpasses the node, the P wave demonstrates a biphasic pattern. The optimal tip position is determined when the P wave amplitude ranges between 50 and 80% of the QRS complex amplitude on the intracavitary ECG (IC-ECG) [21].

The only limitation is that it cannot be utilized in the setting of atrial fibrillation or other arrhythmias where the P wave is absent or uninterpretable [11,22,23].

## 2. Materials and Methods

In 2024, our institute (Luigi Galmarini Hospital—Tradate, ASST-Settelaghi Varese, Italy) enrolled 11 patients with end-stage renal disease and no atrial arrhytmias (Table 1). These patients underwent ultrasound-guided placement of a central venous catheter in the right internal jugular vein. All procedures were performed by the same dedicated team of healthcare providers, including senior surgeons with extensive structured experience in vascular access and nephrologists. The team’s collaborative support and standardized catheter care were achieved by highly trained nursing staff who were proficient in all aspects of catheter maintenance and management.

The correct positioning of the CVC was verified through intracavitary electrocardiography using the MAGELLANO^®^ device. The MAGELLANO^®^ device is a system that detects intracavitary and surface electrocardiographic waveforms with Bluetooth transmission, enabling the accurate placement of central venous access devices by visualizing the P wave and QRS complex. The device consists of an ECG signal acquisition and transmission unit, to which the patient cable with four terminals is connected, enabling real-time monitoring of the electrocardiographic tracing. The ECG signal is detected by an Android^®^ system, smartphone or tablet, in real time. Magellano^®^ consists of a device for capturing and transmitting the ECG signal, to which the 4-terminal patient cable is connected.

**Figure 1:**
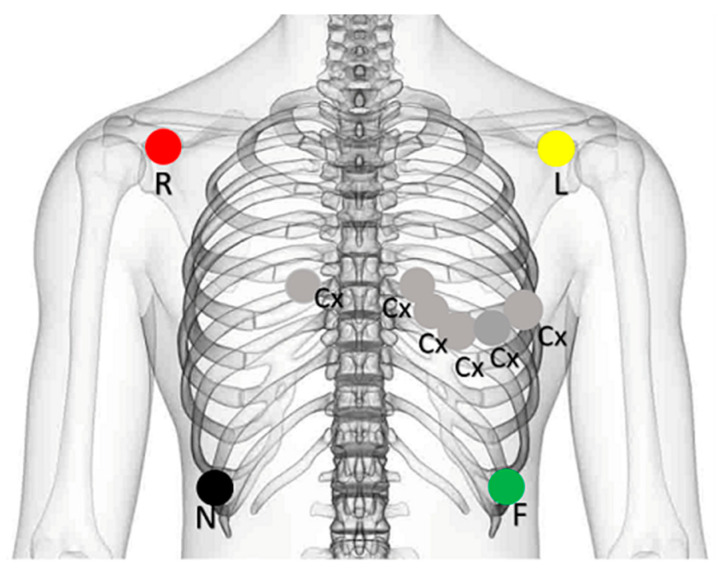
Electrodes placement.

**Figure 2:**
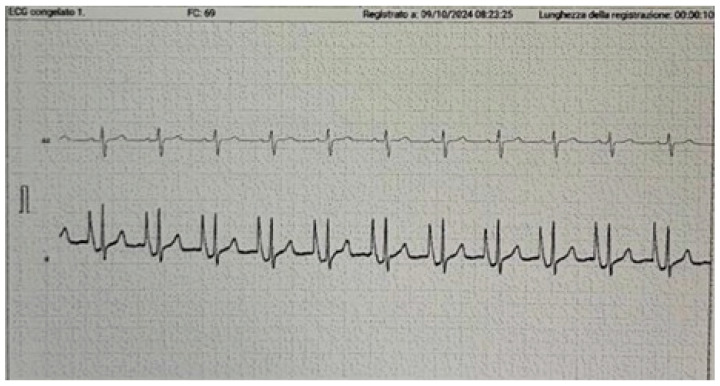
IC-ECG. The top tracing shows the patient’s baseline ECG. The lower tracing shows the P wave maximized at the atriocaval junction, which will then become biphasic and eventually show a deflection.

Intracavitary ECG detects changes in the P wave morphology tracing as the catheter tip approaches the Cavo-Atrial Junction. The catheter, filled with saline solution, acts as an exploring electrode through the distal end. The P wave progressively increases as the catheter approaches the right atrium. At the Cavo-Atrial junction, it reaches its maximum height, then becomes biphasic in the right atrium, and finally completely negative once it has been passed (Figure 1 and Figure 2).

The use of this technique regarding Hemodialysis Catheters does not involve any particularity as these devices are entirely and completely central venous catheters that are commonly used in the clinical setting.

Concurrently, a non-systematic review of the most recent literature was conducted through the PubMed—United States National Library of Medicine database and MDPI references, focusing on the topics of ultrasound-guided placement of central venous catheters and the verification of their proper positioning using intracavitary electrocardiography.

## 3. Results

Before the procedure, a proper skin antisepsis was performed using Iodopovidone. All procedures were carried out under strict sterile conditions, with the utilization of caps, masks, sterile gowns, sterile gloves, and the application of an adequate sterile drape to ensure a safe and controlled environment for the medical intervention.

The same technique was used for all patients, which is described as follows: “Local anesthesia was administered, followed by US-guided venipuncture of the lower third of the RIJV. Using the Seldinger technique, a metal guidewire was inserted up to the RA. A CVC of the Arrow Vector Flow type was then cannulated suprasternally, with subsequent subcutaneous tunneling to the right hemithorax. The tip location was verified using IC-ECG through the Magellano^®^ system. Lung injury was assessed by the US, confirming the presence of pleural sliding. Finally, a flat dressing was applied”. The mean placement time was 28 ± 4 min. Subsequent management of the CVC involved the use of heparin as the locking solution and the use of low molecular weight heparin during the session.

Following the procedure, a chest X-ray in two views was always conducted for further verification of the correct tip location. The radiological assessment confirmed the absence of iatrogenic lung injuries and the proper positioning of the central venous catheter in all 11 cases. This approach demonstrated exceptional efficacy and safety, with no complications recorded. The study’s findings suggest that this technique is a reliable and effective method for central venous catheter placement, providing accurate tip positioning and ensuring patient safety. The utilization of this technique can contribute to improved patient outcomes by minimizing the risk of complications associated with central venous catheterization.

This work represents the first approach of our institute to this methodology and therefore necessarily shows limitations due to the small size of the sample.

## 4. Discussion

While placing RIJV access using the blind technique, clinicians may encounter several challenges due to less prominent anatomical landmarks, particularly in obese patients or those with a swollen/short neck. This can result in an 11% rate of puncture-related complications. To mitigate this inconvenience, multiple studies have demonstrated the clear advantages of using ultrasound guidance for central venous catheterization. Ultrasound visualization of the internal jugular vein and surrounding anatomical structures can significantly improve procedural success rates and reduce the incidence of complications such as arterial puncture, hematoma formation, and pneumothorax when compared to the traditional blind technique. The use of real-time ultrasound guidance has been shown to increase first-pass success, decrease the number of needle passes, and provide a safer approach for central venous access, particularly in challenging patient populations [20]. Subsequently, joint guidelines from the American Society of Echocardiography and the Society of Cardiovascular Anesthesiologists, as well as the European Society of Anaesthesiology and Intensive Care, have strongly recommended the use of real-time ultrasound guidance for placement of right internal jugular vein central venous access in order to evaluate the location of the vessel before performing venipuncture [1].

Utilizing ultrasound guidance for the procedure ensures an overall success rate of 100%, a significant improvement compared to the 88% success rate observed with the landmark technique. Furthermore, the complication rates are also significantly lower when using US guidance, with a decreased incidence of hematoma formation, carotid artery puncture, and brachial plexus irritation, leading to improved patient outcomes and safety [20].

In the past, it was standard practice to obtain an X-ray after the insertion of a central venous catheter. The rationale was to exclude any potential pulmonary complications, such as pneumothorax, and evaluate the correct positioning of the catheter tip [20]. However, this additional imaging procedure was not only costly but also time-consuming and exposed the patient to potentially harmful ionizing radiations. The use of this routine post-procedure chest X-ray has been called into question, as it may not always be necessary and can add unnecessary burden to both the patient and the healthcare system.

In 1989, Serafini adapted a previous technique used for the positioning of ventricular-atrial shunts for draining hydrocephalus and introduced the intracardiac electrocardiogram method [17]. Before the introduction of this technique, malposition complications were frequently reported. However, the placement of catheters with intracardiac electrocardiography demonstrates an extremely high accuracy of 95.8% (8, 15). IC-ECG can determine the correct tip position with 82% sensitivity, 81% specificity, and a positive predictive rate of 95% [9]. The IC-ECG technique allows the operator to establish the exact position of the catheter tip by observing the deflection of the P wave as it advances toward the right atrium [22]. This negative deflection progressively increases in amplitude, becoming biphasic at the level of the sinoatrial node and positive at the ventricle. This permits the placement of catheters in their most suitable position, at the level of the confluence of the superior vena cava in the right atrium, where the P wave has a deep negative deflection similar to the negative spike of the R-wave, allowing for accurate placement of the catheter tip [11,17,18].

Thanks to these techniques, clear advantages can be deduced. As previously stated, the placement of a central venous catheter under ultrasound guidance reduces the risk of vascular and pulmonary complications related to the procedure. Additionally, the correct positioning of the catheter tip, verified by intracavitary ECG, provides immediate feedback during insertion, allows for live and immediate confirmation and permits clinicians to adjust the catheter position in real time, which would not be guaranteed by a radiological check following the procedure. In case of incorrect positioning confirmed radiologically only later, a repetition of the technique may be necessary, from which further iatrogenic risks would arise. The placement of the catheter under ultrasound guidance thus allows for the dynamic recognition of anatomical structures and the correct identification of the right internal jugular vein, and the verification of the correct tip location by intracavitary ECG ensures immediate control of its position, drastically reducing the number of repeated, possibly dangerous procedures that are thus rendered unnecessary. This results in significant benefits, including decreased procedural complications, improved patient safety, and more efficient utilization of healthcare resources.

The intracavitary ECG technique shows an evident ease of use as it can be performed using standard ECG equipment and a saline-filled catheter, making it applicable and accessible in all clinical settings. This ease of use means that it does not require excessively specialized training beyond a knowledge of ECG interpretation and catheter placement skills. The advantages of this technique are also evident when compared to other methods. As previously stated, chest X-ray, while considered the gold standard, has numerous limitations such as the lack of real-time imaging, exposure to radiation, and delayed confirmation of the final catheter position. Ultrasonography is useful for guiding catheter insertion, but it is unable to reliably confirm the final position of the catheter tip at the cavoatrial junction. Transesophageal echocardiography, although extremely accurate, is invasive, not practical for routine use, and requires highly specialized personnel training. Finally, fluoroscopy, while providing real-time imaging, involves radiation exposure and is not always available in all clinical settings.

Therefore, saline-guided ECG represents a highly advantageous technique as it combines the accuracy in confirming the correct position of the central catheter, the absolute simplicity in execution, the lack of radiation exposure, and contained costs compared to other methods.

If an X-ray double-check is necessary, these two techniques are agreed in 93% of cases, with the difference in most cases implying a higher location of the catheter tip on the X-ray image. This is particularly relevant when the X-ray control is taken with the patient in the standing position and during inspiration, as the change in body position and respiratory phase can affect the apparent position of the catheter tip on the radiographic image. The impact of body position and respiratory phase on the apparent catheter tip location highlights the importance of carefully considering these factors when interpreting X-ray images to ensure an accurate assessment of catheter placement [8,13,24].

An ultrasound and intracardiac electrocardiogram-assisted guide wire and catheter insertion allow clinicians to conclude that an X-ray may be omitted when the position of the guide wire and the catheter is confirmed by ECG, thereby reducing radiation exposure for the patient. This technique, when applicable, enjoys near-total accuracy, comparable in the literature to that of transthoracic echocardiography. The only universally stated limitation is represented by the presence of Atrial Fibrillation or further alterations of the heart rhythm that make the P wave absent or unrecognizable. The use of these techniques, that are safe and effective, has made traditional X-ray control obsolete. When the guide wire and catheter positions can be reliably confirmed using ECG, X-ray control is uncessary even to minimize the patient’s radiation exposure [10,23].

## 5. Conclusions

The real-time US guidance has been found to offer several clinically significant advantages, including reducing the risk of failed catheter placement, decreasing the time required for successful vein puncture, lowering the risk of arterial punctures, and minimizing hematoma formation. Placing CVC in the RIJV under US guidance and verifying the tip position using IC-ECG constitute the optimal approach.

This technique is highly accurate, sensitive, and specific. It is also more cost-effective, straightforward to teach and learn, and can be performed without issue in various healthcare settings. Consequently, X-ray control may be safely omitted in more than 90% of cases. A chest radiogram in two views should be obtained when the operator deems it necessary and is mandatory when the P wave is absent or unreadable. Arrhythmias with an absent or unreadable P wave represent the sole limitation of the IC-ECG method.

Intracavitary ECG proves its superiority to other techniques for verifying catheter tip location thanks to its real-time feedback, high accuracy, and reduced exposure to radiation.

When placing a CVA device in non-bedridden patients, it is recommended to position the tip slightly lower (1–2 cm) than the final desired location during the procedure to prevent the catheter from migrating out of the SVC and entering the RA due to changes in patient position.

These statements are supported and ratified by the most recent and authoritative guidelines. Of notable importance is that, by applying this methodology, the verification of the correct positioning of the catheter occurs during the procedure and not afterward, which means that the outdated strategy of positioning a central vascular access approximately and only subsequently verifying its correct localization, with the risk of necessary further measures that an intra-procedural verification would allow to avoid, is no longer acceptable.

A sine qua non condition for the correct execution of this technique is that the operators involved have received proper and specific training. Like every medical procedure, the level of experience of the physicians reduces the risk of complications. Clinicians who routinely place and manage central venous catheters should be well versed in the possible complications of the procedure, particularly in high-risk patients. Proper training, ongoing education, and adherence to evidence-based guidelines can help minimize the risk of adverse outcomes associated with CVC placement and management.This study presents necessary limitations due to the small size of the group, the single-center design, the absence of a control group, and, as it represents the first approach of our institute to this technique, the limited experience in the use of the device.

## Figures and Tables

**Table 1:** Population characteristics and results.

Sex	Male: 6 Female: 5
Indication	End Stage Renal disease
Mean Age	69
Operative Time	28 ± 4 min
Complications	No (0/11)
X-Ray Confirmation	Yes (11/11)

## Data Availability

The original contributions presented in the study are included in the article, further inquiries can be directed to the corresponding author.

## Abbreviations

The following abbreviations are used in this manuscript:

AVF	Arterio-Venous Fistula
RIJV	Right Internal Jugular Vein
US	Ultrasound
CVA	Central Venous Access
CAJ	Cavo-Atrial Junction
SVC	Superior Vena Cava
RA	Right Atrium
IC-ECG	Intracavitary ECG
